# Identifying acute lymphoblastic leukemia mimicking juvenile idiopathic arthritis in children

**DOI:** 10.1371/journal.pone.0237530

**Published:** 2020-08-11

**Authors:** Ninna Brix, Steen Rosthøj, Mia Glerup, Henrik Hasle, Troels Herlin

**Affiliations:** 1 Department of Pediatric and Adolescent Medicine, Aarhus University Hospital, Aarhus, Denmark; 2 Department of Pediatric and Adolescent Medicine, Aalborg University Hospital, Aalborg, Denmark; German Cancer Research Center (DKFZ), GERMANY

## Abstract

**Objective:**

Acute lymphoblastic leukemia (ALL) may present with arthritis implying the risk of being misdiagnosed as juvenile idiopathic arthritis (JIA). The aim of this study was to identify predictors for ALL based on clinical and laboratory information.

**Methods:**

This cross-sectional, retrospective study compared clinical presentation and laboratory results of 26 children with ALL and arthritis versus 485 children with JIA (433 non-systemic, 52 systemic JIA). Using a Bayesian score approach the findings were evaluated by calculating odds ratios (OR) and lnOR as a measure of diagnostic weight.

**Results:**

Distinction on clinical grounds was difficult, as even a high number of joints involved did not exclude ALL. One or more hematologic cell counts were low (Hb <10 g/dL, platelet count <100 x 10^9^/L, neutrophil count < 1.0 x 10^9^/L) in 92% with ALL, 25% with systemic JIA and 10% with non-systemic JIA. Neutropenia and thrombocytopenia had the highest ORs of 128 (95% CI 43–387) and 129 (95% CI 26–638), each giving a diagnostic weight of 4. The estimated risks of ALL were 0.2% with normal cell counts and 9%, 67% and 100% when one, two or three cell lines were affected.

**Conclusion:**

A simple count of cell lines with low counts can serve as a basic diagnostic strategy. Children with tri- or bilinear involvement should be referred to a bone marrow, and those with unilinear involvement a thorough screen for further evidence of ALL (organomegaly, ESR, LDH, uric acid, and blood smear).

## Introduction

Acute lymphoblastic leukemia (ALL) may initially present with arthritis as part of a prodromal stage lasting for weeks or even months, without typical signs of leukemia. Thus, ALL may be misdiagnosed as juvenile idiopathic arthritis (JIA), leading to a delay in proper treatment [1]. JIA is the most common chronic inflammatory joint disease in children with an annual incidence of 15 per 100,000 children in the Nordic countries [2,3]. JIA is defined as persistent arthritis in one or more joints for more than six weeks, with an onset before the age of 16 years and where other causes are excluded [3,4]. Since JIA is a diagnosis of exclusion, and since treatment with corticosteroids may be given, a significant delay in establishing the ALL diagnosis may ensue in children with ALL and arthritis suspected as JIA [5–7].

ALL is the most common childhood neoplasia [8] and the one most frequently presenting with arthropathy (arthralgia and arthritis) at disease onset [9,10]. Arthralgia has been found in 16–20% [1,7,11] and arthritis in 2–10% of children with ALL [1,5,12–14].

In our previous study [1], we compared 53 children with ALL and arthropathy (27 arthralgia, 26 arthritis) versus 233 children with ALL without arthropathy. The children with arthropathy had less clinical and laboratory signs of leukemia and the diagnostic delay was twice as long. Of the children with ALL and arthritis, 88% were misdiagnosed, hereof 26% as JIA. Of these children, 70% received intraarticular corticosteroids before ALL was diagnosed [1]. Corticosteroids may, even when given intraarticularly, relieve symptoms, change the cytology of the bone marrow and reduce the subsequent response to chemotherapy [15].

Previous studies comparing children with ALL and JIA primarily involved children with ALL and musculoskeletal symptoms [7,12,16–19], and only two smaller studies involved children with ALL and arthritis versus JIA [20,21]. Different clinical and laboratory features have been noted, but a useful general approach to differential diagnosis has not been described.

The aim of this study was to identify predictors for ALL using basic clinical and laboratory information for use in daily clinical practice to distinguish between children with ALL and arthritis and children with JIA.

## Material and methods

In this retrospective, cross-sectional study we compared the number of joints with arthritis and laboratory values of children with ALL and arthritis versus children with JIA. All children included in the study had an established diagnosis of pre-B ALL or JIA at the time of inclusion. The diagnosis of ALL was based on bone marrow biopsy and the diagnosis of JIA regarding the International League of Associations for Rheumatology (ILAR) criteria [22]. Laboratory data were collected from diagnosis date +/- one week. The value closest to the diagnosis time was used. We identified the children with ALL and arthritis by review of medical records from all consecutive patients at the age of 1 to 14 years diagnosed with ALL (301 children) at pediatric oncology units at Aalborg University Hospital and Aarhus University Hospital from January 1992 to March 2013. Inclusion criteria were presence of arthritis: swelling within a joint and/or limitation of joint motion with arthralgia.

The children with JIA were recruited from a patient cohort at the pediatric rheumatology unit, Aarhus University Hospital, from January 2000 to December 2014 containing all consecutive children at the age 1 to 16 years and diagnosed with JIA in this period. JIA encompasses seven different subtypes based on the ILAR criteria [22]. Inclusion criteria were systemic, oligoarticular or rheumatoid factor-negative (RF-neg) polyarticular JIA, as these subtypes are at the highest risk of being misdiagnosis with ALL.

We extracted data on age, gender, number of joints with arthritis in addition to laboratory tests on complete blood count, C-reactive protein (CRP), erythrocyte sedimentation rate (ESR), lactate dehydrogenase (LDH), uric acid, IgM rheumatoid factor (RF), anti-nuclear antibodies (ANA), HLA-B27 and blasts in peripheral blood. Quantitative hematological data were categorized using the following definitions: Leukopenia = white blood cell count (WBC) <4.0×10^9^/L, leukocytosis = WBC >20.0×10^9^/L, neutropenia = neutrophil count <1.0×10^9^/L, anemia = hemoglobin <10.0 g/dL (independent of age) and thrombocytopenia = platelet count (PLC) <100×10^9^/L. Cut-off for high levels of LDH were 500 IU/L, for uric acid 0.35 mmol/. IgM-RF was defined as positive when ≥ 30 U/ml. ANA was defined as positive when ≥1:160 and analyzed according to Glerup et al 2017 [23].

### Statistical analysis

Statistical analyses were performed in STATA IC 16.0. Continuous variables are reported with median and range or interquartile range (IQR), categorical variables with percentages. Differences between patient groups were compared using the Mann-Whitney test for continuous variables and Fisher’s exact test for categorical variables. The statistical significance level was set at 5% (p<0.05). Using a Bayesian score approach odds ratios (OR) were calculated to assess the strength of association with ALL, lnOR to get a measure of the weight of evidence in favor of ALL, both with 95% confidence intervals (CI). By rounding off the values to whole integers, they can be used in an additive clinical score showing the amount of evidence in favor of ALL. Further explanation is included in S1 Appendix: lnOR as an additive measure of diagnostic weight of evidence.

Predictive values of the findings were calculated assuming a representation of ALL and JIA at a ratio 13:485 rather than 26:485 as in the study material of two independent cohorts. This corresponds better to an expected prevalence in the test population of approximately 3% (incidence of ALL with arthritis approximately 0.5/10^5^, of JIA 15/10^5^).

## Results

We examined data on 26 children with pre-B ALL and arthritis, i.e. swelling of a joint (65%) and/or restriction of motion (88%) with pain (100%) and compared with 52 children with systemic JIA, and 433 children with non-systemic JIA (hereof 213 with persistent oligoarticular JIA, 96 with extended oligoarticular JIA and 124 with RF-negative polyarticular JIA). The median age was 5.9 years (IQR 5.7) for children with ALL, 9.5 years (IQR 6.5) for those with systemic JIA, and 7.0 years (IQR 8.3) for those with non-systemic JIA. Gender distribution did not differ significantly: 58% (15/26) of the children with ALL were girls versus 42% (22/52) of the children with systemic JIA (p = 0.15), and 68% (158/433) of those with non-systemic JIA (p = 0.19). HLA-B27 was positive in 9% (30/326) of children with JIA, but none of 16 tested children with ALL were positive (p = 0.15). ANA was positive in 42% (204/485) of the children with JIA versus 9% (1/11) with ALL (p = 0.02). Twelve children with ALL were tested for IgM-RF and all were negative. Nocturnal pain was not reported in any of the children with ALL. The number of joints affected did not distinguish children with ALL clearly from those with JIA (**Table 1**).

**Table 1:** Number of joints affected at diagnosis in children with ALL and arthritis, systemic JIA and non-systemic JIA.

	ALL + arthritis n = 26	Systemic JIA n = 52	P	Non-systemic JIA n = 433	P
0 joint	0%	64%	< 0.001	0%	1.000
1 joint	38.5%	13%	0.014	45%	0.328
2–4 joints	38.5%	13%	0.014	35%	0.432
5–8 joints	23%	6%	0.033	13%	0.123
9–28 joints	0%	4%	0.442	7%	0.164

Polyarthritis (≥ 5 joints) occurred in 23% (6/26) of the children with ALL versus 10% (5/52) with systemic JIA (p = 0.10) and 20% (87/433) with non-systemic JIA (p = 0.44). Involvement of >8 joints did not occur in ALL. Of children with systemic JIA, 65% (34/52) did not have arthritis at onset of disease.

**Fig 1:**
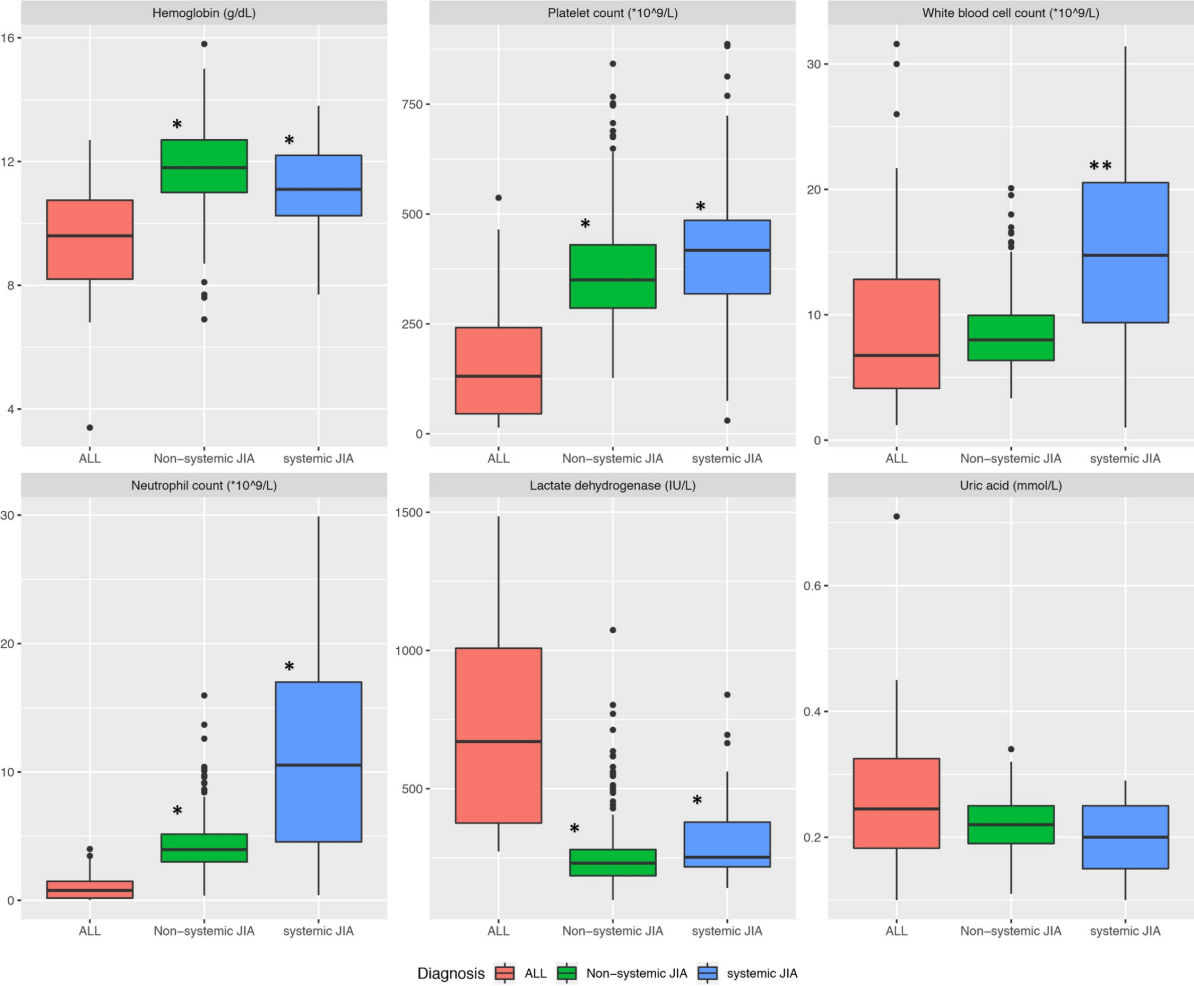
**Box plot comparing laboratory values as median (horizontal line), interquartile range (the box) and total range (vertical line with spots showing outliners) for children with ALL, systemic JIA and non-systemic JIA.** * p-value <0.001 ** p = 0.005.

Hematologic parameters including hemoglobin, platelets, leukocyte and neutrophil counts were significantly lower in children with ALL compared to children with JIA, with the most marked differences in neutrophil and platelet counts as illustrated in the box plot (**Fig 1**).

The ranges of blood counts obtained from the ALL and JIA cohorts overlapped considerably, but ranges specific for ALL—i.e. extending the ranges seen in JIA—could be defined for the three cell lines: Hb < 6.5 g/dL (one case), neutrophil count < 0.35 ×10^9^/L (nine cases) and platelet counts < 30 ×10^9^/L (three cases). When including specific values for LDH ≥ 1100 IU/L (six cases), uric acid ≥ 0.35 mmol/L (four cases) and lymphoblasts in peripheral blood (13 cases) the number of children with laboratory values specific for the ALL increased to 73% (19/26). Organomegaly was not present in any of the remaining children.

CRP values were highest in systemic JIA (median 45 mg/L, range 4–373) versus 15 mg/L (2–159) in ALL (p = 0.004) and 5 mg/L (0.5–288) in non-systemic JIA (p = 0.01). Median ESR did not differ between ALL 62 mm/hr (24–137) and systemic JIA 52 mm/hr (2–130), but ESR was significantly lower in non-systemic JIA: 13 mm/hr (1–117), p-value <0.001.

Abnormal laboratory values occurred most often in the group of children with ALL and arthritis (**Table 2**). We calculated OR with 95% CI and diagnostic weight of evidence in favor of ALL, as derived from lnOR (see S1 Appendix). Except for CRP elevation above 50 mg/L, all findings had statistically significant associations with ALL. Neutropenia and thrombocytopenia were the most distinct findings, strongly associated with ALL with ORs of 128 and 129 and a derived weight of evidence of 4.

**Table 2:** Diagnostic value of laboratory parameters in distinguishing children with ALL and arthritis from children with JIA.

	ALL n = 26	Systemic JIA n = 52	Non-systemic JIA n = 433	Total JIA n = 485	OR (95% CI)	lnOR (SE)	Weight
Anemia, Hb <10 g/dL	62%	19%	9%	10%	4.2 (6.1–33.1)	2.66 (0.43)	2
Thrombocytopenia, PLC <100 ×10^9^/L	35%	4%	0%	0.4%	128.0 (25.6–637.5)	4.85 (0.82)	4
Leukocytosis, Leuk >20 ×10^9^/L	15%	27%	0.2%	3%	5.70 (1.8–18.9)	1.74 (0.60)	1
Leukopenia, Leuk <4.0 ×10^9^/L	23%	8%	0.9%	2%	17.9 (5.7–56.4)	2.88 (0.59)	2
Neutropenia, Neu <1.0 ×10^9^/L	65%	6%	0.9%	1%	129.0 (42.9–387.4)	4.86 (0.56)	4
LDH >500 IU/L	62%	18% (n = 34)	11% (n = 146)	12% (n = 180)	10.0 (4.0–25.4)	2.31 (0.47)	2
Uric acid >0.35 mmol/L	15%	0% (n = 9)	0% (n = 37)	0% (n = 46)	*	*	*
CRP >50 mg/L	19%	47% (n = 49)	5% (n = 350)	13% (n = 399)	1.8 (0.7–5.1)	0.60 (0.52)	0
ESR >50 mm/hr	76% (n = 21)	56% (n = 50)	8% (n = 421)	14% (n = 471)	20.7 (7.3–58.6)	3.0 (0.53)	3

We selected the three most statistically significant and relevant laboratory values and rescaled: neutropenia = 2, thrombocytopenia = 2 and anemia = 1, giving a score from 0–5 **(Table 3A)**. Leukopenia was not included, usually reflecting neutropenia. Addition of raised LDH or urate was not evaluated because values were missing in the majority of JIA cases. Addition of elevated ESR to the score did not improve discrimination. Calculated with a prevalence correction, the estimated risk of ALL increased from 3% for score 1 (anemia) to 40% for score 2 (neutropenia or thrombocytopenia), 67% for scores 3 and 4, and 100% for score 5.

**Table 3:** Two hematological scoring systems to distinguish children with ALL and arthritis from children with JIA. **A.** A weighted count of involved cell lines (anemia 1, neutropenia 2, and thrombocytopenia 2). **B.** A simple count of number of involved cell lines.

	ALL n = 26	Systemic JIA n = 52	Non-systemic JIA	Predictive value* % (ALL:JIA ratio)
**A. Weighted count of involved cell lines**				
0	8%	75%	90%	0.2% (1:430)
1	12%	17%	9%	3% (1.5:47)
2	31%	4%	1%	40% (4:6)
3	31%	2%	0%	80% (4:1)
4	0%	2%	0%	0% (0:1)
5	19%	0%	0%	100% (2.5:0)
**B. Simple count of involved cell lines.**				
0	8%	75%	90%	0.2% (1:430)
1	42%	21%	10%	9% (5.5:53)
2	31%	4%	0%	67% (4:2)
3	19%	0%	0%	100% (2.5:0)

* Calculated with 13 rather than 26 ALL cases in order to get representation closer to that expected from incidence figures (5 ALL with arthritis for every 150 JIA).

Predictive values based on a simple count of involved cell lines are shown in **Table 3B**. In children with ALL, one to three cell lines were affected in 92%; in systemic JIA one or two lines were affected in 25%, and in non-systemic JIA one line was affected in 10%. Calculated with a prevalence correction, the estimated risks of ALL were 0.2% with normal counts and 9%, 67% and 100% when one, two or three cell lines were affected.

## Discussion

Children with ALL occasionally present with arthritis in whom the signs of leukemia may be subtle or missing. The aim of this study was to identify predictors for ALL using basic clinical and laboratory information. Surprisingly, clinical distinction between JIA and ALL is difficult, as even a high number of joints involved does not exclude ALL.

However, laboratory tests were helpful. Hematology counts were significantly lower in children with ALL, and abnormal laboratory values were much more frequent in ALL, with neutropenia and thrombocytopenia showing the highest ORs and diagnostic weights. A simple approach to detect ALL among children with arthritis would be to count the number of cell lines affected. We found this to be useful, since involvement of more than one cell line (observed in 4% of systemic JIA) is rare in children with JIA. We suggest that children with trilinear involvement (predictive value of ALL = 100%), and that those with bilinear involvement (predictive value 80%) should have a bone marrow examination to distinguish between ALL and systemic JIA. In those with unilinear cytopenia (with a predictive value 9%), especially in cases with neutropenia or thrombocytopenia, a thorough screening for ALL is warranted and close clinical follow-up. Screening for ALL should include LDH, uric acid and ESR, examination for organomegaly, blood smear and/or flow cytometry to detect blasts in peripheral blood. However, sensitivity to detect blasts in peripheral blood is lower when the leukocyte count is low, as is often the case with arthritis [24].

Because patients with ALL can occasionally have normal indices during the prodromal phase of the disease, a referral to a hematologist/oncologist should be considered for reasons other than joint pain or a lack of laboratory markers for inflammatory diseases. The literature primarily includes comparison of children with ALL and musculoskeletal symptoms versus children with JIA [6,7,12,16–19,25]. Musculoskeletal pain, most often diffuse bilateral leg pain, among children with ALL is known to be associated with nearly normal hematologic counts, with the risk of misdiagnosis as JIA [9,14,25,26]. Nocturnal pain, thrombocytopenia and leukopenia in different combinations have been proven as highly sensitive and specific markers for ALL [6,7]. None of the children with ALL and arthritis in the present study had nocturnal pain. According to Agodi et al [19], the combination of neutropenia, anemia and elevated LDH demonstrated a 93% sensitivity and a 100% specificity of having ALL. LDH has also been identified as a useful marker for ALL [9,17]. Wallendal et al [20] investigated twelve children with malignancies (eight with ALL) and joint involvement with normal blood count and compared with 24 children with JIA. They found LDH as the most value marker to differentiate. LDH was 2.2 times the normal values in the ALL patients versus 0.8 times the normal value for patients with JIA (P = .004, Mann-Whitney U test). Kirubakaran et al [21] compared 10 children with ALL and arthritis, initially suspected as systemic JIA with 10 age-matched children with systemic JIA. Lymphocytosis and thrombocytopenia (each occurring in 70% with ALL and none of the children with JIA) were found to be the most helpful laboratory tests to distinguish between ALL and JIA.

Antinuclear antibodies are not specific for rheumatic disease and may be positive in healthy children as well as in children hospitalized for various reasons [7,25,27]. In the present study, 4% of the children with ALL were ANA positive, similar to ANA frequency in healthy children (2–9%) [28,29]. Previous studies cautioned the use of ANA, because it has been found in 16–22% of children with non-rheumatic musculoskeletal symptoms [7,30–32]. However, some studies used a low cut-off level of 1:20, which might result in false positive results [9].

Limitations to this study include that it is a retrospective study of two separate and independent patient cohorts, with inherent limitations in design and the risk of selection bias. Secondly, the analysis is based on a small cohort of children with ALL with broad confidence intervals for frequencies of abnormal laboratory findings. Thus, the diagnostic weights of some findings may be overestimated. We tried to counteract this by adjusting estimated weights downwards. Given the low rate of ALL with arthritis, a future study with a prospective design necessitates the collection of data from several countries. To our knowledge, this study presents the biggest sample of ALL and arthritis compared to JIA to date. The data material, mainly laboratory values, is reliable with a low risk of information bias.

Finally, the construction of the Bayesian score may fail to adequately address interdependence between findings. In practice, however, such simple scores may be more useful than logistic regression analyses that are complex and often associated with the risk of overfitting the test population. The Bayesian score approach has previously been used successfully to construct a prognostic score for Immune Thrombocytopenia in children [33] and for prediction of bacteremia in febrile neutropenia during ALL therapy [34]. In the present study, the only advantage of the score over a simple count of affected cell lines was to divide children with unilinear involvement into a lower-risk and a higher-risk group.

In conclusion, we have found that low hematology counts are more frequent in children with ALL and arthritis than in children with JIA, but in most cases not so low that JIA can be excluded. A simple count of the number of affected cell lines may help detect ALL cases and serve as a basis for a diagnostic strategy. The Bayesian score approach clarify that neutropenia and thrombocytopenia had the highest diagnostic weight of 4 and the primary advantage of the score over a simple count of affected cell lines was to divide children with unilinear involvement into a lower-risk and a higher-risk group. We recommend that children with tri- or bilinear cytopenia should be referred to a bone marrow, and those with unilinear involvement a thorough screen for further evidence of ALL (organomegaly, ESR, LDH, uric acid, and blood smear). The utility of this approach needs to be tested prospectively in an unselected cohort of children with presumed JIA.

## Supporting information

S1 Appendix lnOR as an additive measure of diagnostic weight of evidence. (DOCX)

S1 File (XLSX)
