# Supplementary material for: Identifying acute lymphoblastic leukemia mimicking juvenile idiopathic arthritis in children
Source: PLoS One. 2020 Aug 11;15(8):e0237530. doi: 10.1371/journal.pone.0237530 (PMC7418991; doi:10.1371/journal.pone.0237530)
Supplement: S1 Appendix — (DOCX) [file pone.0237530.s001.docx]

**S1 Appendix: lnOR as an additive measure of diagnostic weight of evidence**

Bayes theorem can be stated in terms of Odds and likelihood ratios (LR). It then simply states that *posttest Odds = pretest Odds x LR of the observed test result,* positive (LRpos) or negative (LRneg)*.* For a sequence of tests, assuming independence between the results, pretest Odds are multiplied sequentially with the LRs of the observed test results: *posttest Odds = pretest Odds x LR_1_ x LR_2_ x LR_3_ …..*

In the evaluation of a profile of test results, a patient with all tests negative - i.e. with no evidence of the disease in question - can be used as reference. If three tests have been performed:

*(1) Odds(neg-neg-neg) = pretest Odds x LR_1_neg x LR_2_neg x LR_3_neg*

For a patient with e.g. a positive, a negative and a positive test:

*(2) Odds(pos-neg-pos) = pretest Odds x LR_1_pos x LR_2_neg x LR_3_pos_3_*

Dividing (2) with (1) it is seen that:

*(3) Odds(pos-neg-pos) = Odds(neg-neg-neg) x (LR_1_pos/LR_1_neg) x (LR_2_neg/LR_2_neg) x (LR_3_pos/LR_3_neg).*

Since LRpos/LRneg is equal to the Odds ratio (OR) of the positive result the formula can be rewritten:

*(4) Odds(pos-neg-pos) = Odds (neg-neg-neg) x OR_1_ x 1 x OR_3_*

showing that Odds relative to Odds in the reference patient without any evidence of the diagnosis have been increased by the product of ORs for all positive test results. This factor is independent of the prevalence of disease in the test population, i.e. pretest Odds, and therefore can be applied in any population.

The multiplicative formula (4) can be converted into a practical additive format by logarithmic transformation:

*(5) lnOdds(pos-neg-pos) = lnOdds(neg-neg-neg) + lnOR_1_ + 0 + lnOR_3_.*

It follows that lnOR can be considered a measure of the weight of evidence in favor of the diagnosis supplied by a positive test result. The total amount of evidence accumulated in a test sequence can be determined by adding up lnORs of all positive results.

Thus, additive clinical scores can be constructed using lnORs, selecting a limited number of presumably independent tests. The lnORs are rounded off to get weights as whole integers, rounding down to counteract interdependence. The weights may be rescaled to get numbers as small as possible, giving the finding with the smallest weight the value 1.

.

----------------------------------------------------------------------------------------------------

Odds = (probability that the disease is present) : (probability that the disease is absent)

Likelihood Ratio (LR) = (frequency of test result when disease is present) : (frequency when absent)

Odds ratio (OR) = LRpos/LRneg.

lnOR = the natural logarithm to OR. Log to base 10 or to base 2 can also be used in the logarithmic transformation if preferred. log_2_OR has a simple meaning: the number of times odds in favor of disease are doubled by the positive test result.
